# Mastery, perceived stress and health-related behaviour in northeast Arnhem Land: a cross-sectional study

**DOI:** 10.1186/1475-9276-5-10

**Published:** 2006-09-27

**Authors:** Mark Daniel, Alex Brown, J Garnggulkpuy Dhurrkay, Margaret D Cargo, Kerin O'Dea

**Affiliations:** 1 Département de médecine sociale et préventive, Université de Montréal, Québec, Canada; 2 Menzies School of Health Research, Darwin, Northern Territory, Australia; 3 Yalu Marnggithinyaraw Centre, Galiwin'ku, Elcho Island, Northern Territory, Australia; 4 Psychosocial Research Division, Douglas Hospital Research Centre, McGill University, Canada; 5 Department of Medicine, St. Vincent's Hospital, The University of Melbourne, Australia; 6 Canada Research Chair for Biopsychosocial Pathways in Population Health, Centre de Recherche du Centre Hospitalier de l'Université de Montréal (CHUM) – Hôtel-Dieu, Édifice Saint-Urbain, Axe santé des populations, 3875 rue Saint Urbain, Montréal, Québec H2W 1V1, Canada

## Abstract

**Background:**

Indigenous peoples in Australia are disadvantaged on all markers of health and social status across the life course. Psychosocial factors are implicated in the aetiology of chronic diseases and in pathways underpinning social health disparities. Minimal research has investigated psychosocial factors and health in Indigenous peoples. This study evaluated associations between mastery, perceived stress, and health-related behaviour for a remote Indigenous population in Australia.

**Methods:**

Complete data on mastery (the degree to which individuals feel in control of their lives), perceived stress, physical activity, and fruit and vegetable consumption were obtained for 177 participants in a community-based chronic disease risk factor survey. Psychosocial questionnaires were completed as an option during community screening (response rate = 61.9%). Extensive consultation facilitated the cross-cultural adaptation of measures.

**Results:**

Mastery was inversely correlated with perceived stress measures (*p *< 0.009): recent stress, *r *= -0.47; chronic stress, *r *= -0.41; and youth stress, *r *= -0.30. Relationships between mastery and behaviour varied according to age group (<25 or ≥25 years) for physical activity (*p *= 0.001) and vegetable consumption (*p *= 0.005). Individuals aged ≥25 years engaging in ≤2 bouts of physical activity/week had lower mastery than individuals engaging in ≥3 bouts/week, with means (95% CI) of 14.8 (13.7–15.8) and 17.1 (15.3–19.0), respectively (*p *= 0.026). Individuals aged ≥25 years eating vegetables ≤3 times/week had lower mastery than those eating vegetables ≥4 times/week (*p *= 0.009) [means 14.7 (13.8–15.5) and 17.3 (15.5–19.1), respectively]. Individuals <25 years engaging in ≤2 bouts of physical activity/week had greater mastery than individuals engaging in ≥3 bouts/week (*p *= 0.022) [means 17.2 (15.2–19.2) and 13.8 (11.9–15.7), respectively]. For men ≥25 years and women ≥15 years, mastery was inversely related to age (*p *< 0.002). Men <25 years had less mastery than women of equivalent age (*p *= 0.001) [means 13.4 (12.1–14.7) and 17.5 (15.3–19.8), respectively].

**Conclusion:**

Consistent with previous research, this study provides additional support for a link between mastery and health-related behaviour, and extends evidence of this association to a remote Indigenous population. Mastery's association with perceived stress, its age-specific association with health behaviour, and findings of low mastery amongst young men, highlights a need for life course research accounting for contextual factors affecting Indigenous peoples.

## Background

Socioeconomic status (SES) is a profound and consistent predictor of health status at both the population and individual level [1], between and within countries across the globe [2]. Despite regularity in patterns of health inequality across SES strata, the means by which disadvantage incurs risk of illness remain inadequately understood. This is not to suggest that little can be done to alleviate health inequalities without a thorough understanding of the biopsychosocial pathways linking social adversity to health disadvantage. Evidence of widening socioeconomic inequalities [3] mandates consideration of collective failures to apply what is already known, or deficits in awareness of more fundamental drivers of disparity and an ability to influence these. This seems particularly poignant when considering the health disadvantage of Indigenous and other marginalised populations, where ill health can be seen as a function of larger forces that impinge *on *specific sub-populations, rather than as a problem *of *these sub-populations.

Indigenous peoples in Australia are profoundly disadvantaged on virtually all markers of health and socioeconomic status across the life course [4,5]. Much of the epidemiological and policy landscape of Australia has supported the "social determinants" perspective of health as a framework through which to understand such disparity. The cultural and gender-specific determinants of behaviour, treatment, health awareness, prevention and causation have yet to be determined, however. Prevention efforts from the individual level to the societal level have not thus far incorporated into practice anything beyond a general understanding of the basis of Indigenous health disadvantage, or attempted to make explicit the filters of such effects.

Psychosocial factors are relevant to understanding how risk conditions affect cognition and thus health through behavioural and biological responses [6]. Such measures are receiving growing acceptance as contributors to disease aetiology [7], and as modifiers and mediators of social health disparity [8]. Support for a psychosocial explanation for health inequalities is most often seen for chronic illness and outcomes including cardiovascular reactivity [9] and mortality [10], survival after myocardial infarction [11], perceived health [12], all-cause mortality [13] and various psychopathological and emotional states [14]. *Control*, operationalised as mastery [15], self-efficacy [16], or locus of control [17], has emerged as a central integrating concept in health research, and as a psychosocial resource in the aetiology of health and disease [18]. A growing literature attests to a connection between social disadvantage, nominal control, risk behaviour and morbidity [19,20]. Chronic low hope for influencing conditions of living is a strong predictor of morbidity and mortality [21], whereas chronic instability and disorder predict psychological distress and poor health behaviour [22,23]. Used as an explanation for how ethnicity, race, or SES relate to health through the quality of experience in one's environ, psychosocial factors have the potential to capture feelings of oppression and alienation relevant to marginalised peoples.

Minimal empirical research has been published on psychosocial factors in Indigenous Australians, and quantitative evidence of a link between psychosocial factors and health has not been reported. Studies amongst Canadian First Nations have documented associations between mastery, health behaviour, and cardiovascular risk [24,25], but such groups have had a longer period of westernisation than Indigenous peoples in Australia. It is important to know if relationships observed for disadvantaged, and primarily non-Indigenous populations elsewhere, apply also to Indigenous Australians who have undergone a recent, rapid transition to westernised living. This study sought to undertake culturally-sensitive measurement of psychosocial factors and to test relationships between mastery, perceived stress, and health behaviours including recreational physical activity and vegetable and fruit consumption, in a remote Indigenous settlement in Australia.

## Methods

Persons surveyed were volunteers for a community-based chronic disease risk factor screening initiative among Yolngu people residing in and around Galiwin'ku settlement, Elcho Island, of northeast Arnhem Land in the Northern Territory of Australia. This population is isolated and, as recently as 1985, people in this region lived a traditionally-oriented lifestyle with no risk factors for diabetes or cardiovascular disease [26].

Persons 15 years of age and older were targeted for screening. Participants gave their written, informed consent. The protocol was reviewed and clearance provided by the Top End Human Research Ethics Committee with input from an Indigenous Sub-Committee. Menzies School of Health Research, in Darwin, Northern Territory, was responsible for the study.

### Population and setting

Galiwin'ku is a remote Aboriginal settlement. It is reachable only by small aircraft or by boat. For the screening initiative conducted from 2001–2002, the population base was determined by household survey, with enumerators conducting door-to-door visits. The adult population aged 15 years and older numbered 751. Yolngu Matha is the lingua franca, with English spoken as a non-maternal language. Welfare entitlements are the primary source of income. Resources in the settlement include a school, a food and general store managed by a Yolngu organisation, a council made up of clan representatives, resident police officer, and two take-out food outlets. A local health centre services the settlement, with health care provided by general practitioners, Aboriginal health workers and resident remote area nurses. Climatic conditions typify the tropical, monsoonal regions of north Australia. The lifestyle of Yolngu is a blend of traditional and now more western ways of living (poor diet and physical inactivity).

### Screening procedures and participation

An extensive course of consultation was enacted prior to and during the screening procedures. Community members and groups had input into research activities, including the format and content of questionnaires and consent forms, site and organisation of the screening, timing of visits from researchers, and administration of questionnaires in local language. Community input was through the Yalu Marnggithinyaraw Clan Management Committee.

Recruitment involved an extensive campaign to encourage adult residents, 15 years and older, to be screened for diabetes, cardiovascular disease, and risk factors. Screening activities, reported elsewhere [27], involved: provision of a fasting blood sample for measurement of lipids and glucose; anthropometric measurements; and a survey with demographic and behavioural components. For the base population of 751 adults, 595 persons (79.2%) completed at least one part of the screening protocol with this sample representative of the overall distribution of age and sex. Variation existed in data coverage; *e.g*., blood samples were provided by 339 individuals. Psychosocial questionnaires were offered as an option during screening.

### Psychosocial measurement: cross-cultural adaptation

The psychosocial questionnaire was developed by adapting measures used in Indigenous health research elsewhere. Measures were chosen for their brevity, cultural salience, use in divergent populations, and known reliability and validity. All questions were translated into Yolngu Matha after revisions to facilitate improved comprehension. Questionnaire adaptation was assisted by interviews with 15 Yolngu (3 men, and 12 women). Translation was followed by back translation into English. These steps were repeated several times over until reasonable consistency was achieved. The Yalu Clan Management Committee approved all measures. An iterative process of pre-testing was initiated following cross-cultural adaptation. Scale response options were reduced from five to three possibilities ("yes," "somewhat," or "no"). A story was drafted and shared with residents to explain the psychosocial survey, why feelings are central to health, and why researchers wished to ask about emotions.

As an optional component of screening, the response rate for completion of psychosocial surveys was less than for other parts of the screening protocol. Home visits were undertaken to improve representation of individuals who did not complete the questionnaire at the screening site. Of 210 psychosocial questionnaires collected (representing 61.9% of the 339 individuals providing behavioural and clinical data), 56 were obtained via home visit.

### Psychosocial and behavioural measures

*Mastery *was measured as an adaptation of the 7-item Likert scale for which construct validity has been confirmed [15]. The Mastery scale is widely cited in the literature on control and has been utilised with First Nations in Canada [24,25]. Respondents rate their level of agreement with seven statements such as, "I can do just about anything I really set my mind to." Scores were summed, multiplied by a factor of ten, and the product divided by the number of items to yield an average response (maximum = 30). Cronbach's α for the translated scale used with the Yolngu was 0.76. The average inter-item correlation was 0.31 (range: 0.14–0.42). Item-total correlations ranged from 0.44–0.58.

A subset of individuals responded to questions (from the Canadian National Population Health Survey) about chronic stress, recent stress and youth stress. Such questions were part of the initial survey but were later omitted, given community concern about numbers of items. All measures were scored as the number of affirmative responses. *Chronic stress *was measured using eight questions about chronic stress, such as, "Is there someone close to you who is in bad health and may die?" *Recent stress *was measured using three questions about recently experienced stressful events, such as, "Have you experienced an increase in arguments with anyone close to you?" "*Youth stress *was measured retrospectively using four questions about stressful events during one's youth, such as "Did something happen that scared you so much that you thought about it for years after?" For stress-related measures, Cronbach's α and average inter-item correlations were: chronic stress, α = 0.70, *r *= 0.22 (*n *= 63); recent stress, α = 0.81, *r *= 0.64 (*n *= 63); and youth stress, α = 0.95, *r *= 0.93 (*n *= 51).

The frequency of recreational physical activity and of vegetable and fruit consumption for the four weeks preceding the survey was assessed as part of the standard screening protocol via questions used earlier for surveys in Aboriginal Australian communities [28]. Response scales for physical activity were: none; 1–2 times/week; 3–4 times/week; and >4 times/week. Response scales for frequency of fruit and vegetable consumption, assessed separately, were: 0–1 times/week; 2–3 times/week; 4–6 times/week; and everyday. To conserve statistical power, responses to the four-category ordinal scales for behavioural frequency were collapsed into two levels combining the two lower and the two upper categories, respectively.

### Statistical analysis

Thirty-three individuals provided incomplete surveys missing key demographic, behavioural or psychosocial data that could not reasonably be imputed. Hence, the effective sample size was 177 persons. Analyses of descriptive characteristics comparing men to women were performed using *t*-tests and *z*-tests for continuous data and proportions, respectively. Analysis of variance was performed to assess age- and sex-related variation in mastery. Age was grouped into four strata, 15–24.9 years, 25–34.9 years, 35–44.9 years and 45+ years, representing 25%, 26%, 27% and 22% of respondents.

Inferential analyses of relationships between mastery and behaviour were performed by maximum likelihood regression, stratified for age <25 years and age ≥25 years, controlling for age and sex. A preliminary analysis indicated that relationships between mastery and behaviour varied according to age group for vegetable consumption (Likelihood ratio [LR] χ^2 ^= 12.8, 3 df, *p *= 0.005), physical activity (LR χ^2 ^= 13.2, 3 df, *p *= 0.001), but not fruit consumption (LR χ^2 ^= 5.2, 3 df, *p *= 0.15). Associations between mastery and all three behaviours were similar for the three upper age groups but had a consistently different pattern for the low age group, 15–24.9 years.

Two approaches were used to determine relations between mastery and perceived stress, (i) Spearman rank-order correlation coefficients, and (ii) standardised β coefficients adjusted for age and sex. For regression analyses, mastery and chronic stress were specified as dependent variables, and recent stress and youth stress specified as explanatory variables. Chronic stress was normally distributed, mastery closely approximated a normal curve, but recent stress and youth stress were not normally distributed. The nonparametric Spearman correlation coefficient was calculated together with standardised β coefficients (ranging from 0–1) to enable assessing the similarity of age- and sex-adjusted parametric measures of association with non-parametric coefficients, thus enabling comparisons of associations for all pairs of psychosocial variables.

## Results

### Participant characteristics

Respondents (*n *= 177) ranged in age from 15–75 years with the mean (standard deviation [SD]) 35.6 (13.4) years. Mean age and the distribution of age did not differ between sexes. One-third of the sample had access to an automobile and one-third access to a working telephone in their home. Two-thirds of respondents had a working refrigerator in their home (Table 1). Twice as many women as men reported having achieved an education at or beyond a Year 10 equivalent (*z *= 4.4, *p *< 0.0001). Half the respondents were daily smokers; slightly more men than women smoked (*z *= 2.2, *p *= 0.029). Neither education nor smoking was associated with mastery. Mean mastery (95% CI) was 14.8 (13.5–16.1) for respondents with less than a Year 10 education and 15.2 (13.7–16.7) for those with a Year 10 education or greater (*p *= 0.69). Mean mastery was 15.3 (14.5–16.0) for smokers and 14.8 (13.7–15.8) for non-smokers (*p *= 0.45). Eleven percent of respondents with psychosocial data were classified as having diabetes, agreeing with 12% so identified of the 339 persons screened [27]. Characteristics of respondents providing mastery data did not differ from those who did not provide mastery data. Descriptive statistics for the four psychosocial measures are given in Table 2.

**Table 1:** Descriptive characteristics, according to sex, for Indigenous respondents

	Men		Women		All (pooled)	
	*n *= 80		*n *= 97		*n *= 177	
	mean	(95% CI)*	mean	(95% CI)*	mean	(95% CI)*
			
Age (years)	33.7	(30.4 – 36.9)	37.1	(34.7 – 39.5)	35.6	(33.6 – 37.5)
Education, completed Year 10 (%)	25.0	(16.4 – 35.3)	58.8	(48.8 – 68.2)	43.5	(36.3 – 50.9)
Access to a car on regular basis (%)	31.3	(21.8 – 42.0)	29.9	(21.4 – 39.6)	30.5	(24.1 – 37.6)
Telephone available in home (%)	26.3	(17.5 – 36.7)	44.3	(34.7 – 54.3)	36.2	(29.3 – 43.4)
Working refrigerator in home (%)	68.8	(58.0 – 78.2)	72.2	(62.6 – 80.4)	70.6	(63.6 – 77.0)
Daily cigarette smoking (%)	58.8	(47.7 – 69.1)	41.2	(31.8 – 51.2)	49.2	(41.8 – 56.5)
Diabetes mellitus (%)^†^	8.8	(3.9 – 16.5)	13.4	(7.7 – 21.3)	11.3	(7.2 – 16.6)
Body mass index (kg/m^2^)	22.2	(21.2 – 23.2)	23.0	(21.9 – 24.1)	22.6	(21.9 – 23.4)
Waist-to-hip girth ratio	0.94	(0.91 – 0.96)	0.92	(0.91 – 0.94)	0.93	(0.92 – 0.94)

*95% confidence interval, exact mid-*p *confidence interval given for proportions; ^†^Determined by fasting glucose screening

**Table 2:** Psychosocial measures and their distributions for Indigenous respondents

	*n*	range	mean	SD^†^	95% CI^‡^	25^th ^percentile	50^th ^percentile	75^th ^percentile
Mastery	177	0–30	15.2	4.24	14.6–15.8	12.9	14.3	17.2
Chronic stress*	79	0–8	4.99	1.94	4.6–5.4	4.0	5.0	6.0
Recent stress*	79	0–3	1.30	1.16	1.0–1.6	0.0	1.0	2.0
Youth stress*	65	0–4	0.94	1.04	0.7–1.2	0.0	1.0	2.0

*Stress measures were removed from the measurement protocol halfway through the survey on the basis of community concerns about excessive numbers of items; ^†^standard deviation; ^‡^95% confidence interval

### Mastery in relation to age and sex

**Figure 1:**
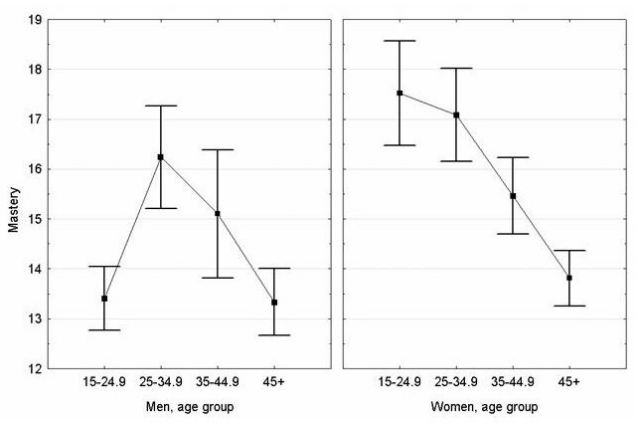
Mean mastery (± standard error) according to age group and sex (*n *= 177).

Mastery varied according to age group and sex (*F*_7,169 _= 3.5, *p *= 0.002). Young men aged 15–24.9 years had less mastery than women of equivalent age, with means (95% CI) of 13.4 (12.1–14.7) and 17.5 (15.3–19.8), respectively (*p *= 0.001). For women, mastery was inversely related to age, with a steady decline across age groups from its peak value observed for those 15–24.9 years of age (*F*_3,93 _= 3.3, *p *= 0.023) (Figure 1). Men were characterised by an inverse U-shaped relationship between mastery and age (*F*_3,76 _= 2.7, *p *= 0.052). For participants aged ≥25 years, the relationship between mastery and age was no different for men than for women (Figure 1). Treating age as a continuous variable, males and females aged ≥25 years were characterised by an inverse linear correlation between mastery and increasing age: *r *= -0.29, *p *= 0.032 for men (*n *= 51); and *r *= -0.30, *p *= 0.001 for women (*n *= 82).

### Mastery and health-related behaviour

Behavioural data were available for 93–99 of 133 respondents aged ≥25 years, and for 42–44 of 44 respondents aged <25 years (*n *varies with the measure). Individuals with behavioural data did not differ from those for whom such data were not available; mastery, age, and proportions for categorical variables were all within the 95% confidence intervals for the overall sample. Relationships between behaviour and mastery did not vary according to sex.

For individuals ≥25 years, engaging in two or fewer bouts of physical activity per week was associated with lower mastery than engaging in three or more bouts per week, adjusted for age and sex (LR χ^2 ^= 4.4, 1 df, *p *= 0.026) (Table 3). Similarly, individuals eating vegetables three or fewer times per week had less mastery than those eating vegetables four or more times per week (LR χ^2 ^= 6.8, 1 df, *p *= 0.009). While not statistically significant, mastery was slightly lower for individuals eating fruit three or fewer times per week compared to those eating fruit four or more times per week (LR χ^2 ^= 2.0, 1 df, *p *= 0.153) (Table 3).

**Table 3:** Relations between mastery and health-related behaviour, according to age group, for Indigenous respondents

	Age less than 25 years					Age greater than or equal to 25 years				
		
	*n*	mean	SE*	95% CI^†^	*p*-value^‡^	*n*	mean	SE*	95%CI^†^	*p*-value^‡^
Vegetable consumption										
Three or fewer times/week	27	15.6	0.69	14.2–16.9	0.345	75	14.7	0.45	13.8–15.5	0.009
Four or more times/week	17	14.3	1.24	11.9–16.7		24	17.3	0.93	15.5–19.1	
Fruit consumption										
Three or fewer times/week	25	15.9	0.70	14.5–17.2	0.131	73	14.9	0.45	14.1–15.8	0.153
Four or more times/week	19	14.0	1.04	12.0–16.0		26	16.3	0.95	14.4–18.2	
Physical activity										
Two or fewer times/week	17	17.2	1.02	15.2–19.2	0.022	69	14.8	0.52	13.7–15.8	0.026
Three or more times/week	25	13.8	0.97	11.9–15.7		24	17.1	0.94	15.3–19.0	

*standard error; ^†^95% confidence interval; ^‡^likelihood χ^2 ^test with 1 df for age stratum-specific difference in mastery between higher and lower frequency of behaviour, controlling for age and sex

For individuals <25 years, adjusted for age and sex, engaging in two or fewer bouts of physical activity per week was associated with greater mastery than engaging in three or more bouts per week (LR χ^2 ^= 5.2, 1 df, *p *= 0.022) (Table 3). While not statistically significant, mastery was marginally higher among individuals consuming vegetables three or fewer times per week (LR χ^2 ^= 0.9, 1 df, *p *= 0.345) and among those consuming fruit three or fewer times per week (LR χ^2 ^= 2.3, 1 df, *p *= 0.131), relative to a consumption of four or more times per week (Table 3).

### Mastery and perceived stress

Mastery was inversely correlated with perceived stress measures, and positive intercorrelations existed between stress measures (Table 4). Standardised β coefficients for age- and sex-adjusted associations between mastery and stress measures were consistent with Spearman coefficients representing the equivalent relationships. The strength of relations between mastery and stress was moderate: recent stress, *r *= -0.47; chronic stress, *r *= -0.41; and youth stress, *r *= -0.30.

**Table 4:** Inter-correlations and standardised β coefficients for association between mastery and perceived stress measures*

		Mastery	Recent stress	Chronic stress
		
Recent stress	*n *for association	79		
	Spearman *r*	-0.47		
	*p*-value (for *r*)	<0.0001		
	β (standard error)	-0.44 (0.11)		
	*p*-value (for β)	0.0001		
Chronic stress	*n *for association	79	79	
	Spearman *r*	-0.41	0.57	
	*p*-value (for *r*)	0.0001	<0.0001	
	β (standard error)	-0.41 (0.11)	0.51 (0.09)	
	*p*-value (for β)	0.0004	<0.0001	
Youth stress	*n *for association	65	65	65
	Spearman *r*	-0.30	0.46	0.38
	*p*-value (for *r*)	0.017	0.0001	0.002
	β (standard error)	-0.33 (0.11)	0.45 (0.11)	0.47 (0.11)
	*p*-value (for β)	0.009	0.0001	0.0001

*β coefficients are adjusted for age and sex

For the 65–79 respondents providing perceived stress data before such questions were removed from the survey, mean age and the distribution of sex did not differ from the overall sample. Relationships between mastery and perceived stress measures did not vary according to age group or gender.

## Conclusion

The data reported here indicate that for Yolngu people in north-eastern Arnhem Land, mastery (i) has age-specific relationships with health behaviour and (ii) is negatively related to perceived recent stress, chronic stress and youth stress. A causal relationship would support action on factors underlying perceived stress as potentially modifiable determinants of mastery. Some such exposures are most likely heterogeneous at the level of the individual ("random stressors") with still others homogenous within (but not necessarily across) disadvantaging environments ("systemic stressors") [29]. The living conditions that define the population surveyed – limited access to transportation, communications and means of food storage, and a low prevalence of even basic education – indicate unequivocally the extent of environmental disadvantage that applies in this setting. While some persons because of their unique experiences may perceive greater stress or lower mastery, there is a need to view the distribution of stressors and stress responses as a function of forces acting on the population as a whole [30].

Additional factors besides perceived stress no doubt influence mastery, and need to be elucidated. Nonetheless, mastery would seem to have cross-cultural utility in linking untoward perceptions that may reflect the disadvantaging experiences of Indigenous people, to actual health behaviour. Little empirical research has targeted mastery or "control" in seeking to understand Indigenous health in a context of westernisation and disadvantage. Our findings that mastery was positively related to health behaviour for individuals ≥25 years of age, while being negatively related to health behaviour for those <25 years of age, indicate that the health correlates of mastery are not invariably positive. Such results suggest that, depending on age or maturity, health salience, or perceived health, mastery may be expressed in different ways. A broader, more positive influence of mastery consistent with findings amongst individuals ≥25 years of age is indirectly supported by a study showing improved health status for Indigenous Australians who relocated to traditional lands relative to counterparts in regional centres [31]. Collectively, homelands dwellers have autonomy, an elevated capacity for self-determination, and less environmental stress. Therefore, at the population level, mastery, conceptually linked to Indigenous people's connection to traditional lands, offers explanatory as well as preventive salience within the context of this analysis.

Used to explain how health is affected by the quality of experience in one's environment, mastery has the potential to contextualise the legacy of colonisation experienced by Indigenous communities and to capture feelings of alienation relevant to marginalised peoples. Alienation in this sense can be understood as a dissociation of people from meaningful work, their social collectivities, or their own identities, or of being distanced from power and resources that may enable self-determination in political, economic, and social settings [32]. Such cognitions may be more common or salient among individuals aged 25 years or more. This broader application of control to health and health-related behaviour reaches beyond past research on job demands and job decision latitude in work environs [33,34]. It is consistent, however, with the growing body of empirical research attesting to important connections between social disadvantage, risk behaviour and morbidity [35-38].

Evidence is accumulating that life experiences and social arrangements, one's place in the social context, or perceived stresses of living, embed themselves in cognitions reflected by psychosocial characteristics and biology over the life cycle [39,40]. This study extends such knowledge to Indigenous Australians who have undergone a recent shift to westernised living in a geographically isolated area. The finding among persons ≥25 years of age that mastery is associated with physical activity and vegetable (but not fruit) consumption has not previously been reported for an Indigenous population in Australia. A lack of association with fruit may reflect its greater popularity and availability in the community. Such relations are consistent with research indicating that mastery is related to behavioural risk factors for First Nations in Canada [24,25]. As depression and hopelessness predict for non-Indigenous populations the development of atherosclerosis [7], type 2 diabetes [41] and coronary heart disease [42], mastery may well be related through behaviour to the development of disease in Indigenous peoples. Few longitudinal studies have been mounted, none featuring psychosocial measures.

That mastery was inversely associated with age for women of all age groups and for men 25 years and older (Figure 1), implies age-dependant decreases in mastery, a phenomenon that for which the literature offers little insight. Most studies on mastery have simply controlled for age. For Indigenous Australians, mastery may reflect different facets of psychological wellbeing at different stages of life, and the apparent decrease may not be pathogenic. Other explanations beyond age include period or cohort effects, but our cross-sectional design precludes assessing the presence of these. Certainly, the finding that higher mastery at younger ages coincides with negative, rather than positive, health behaviour supports longitudinal research on psychosocial status, its evolution and correlates. Given the social and cultural construction of identity, role and place across the life-course, the process of aging may be an important contributor to and manifestation of psychological wellbeing, or of one's sense that they are in control of the factors affecting their lives, opportunity, health, and community wellbeing.

A noteworthy finding of this study was low levels of mastery among men between 15–24 years. Young Indigenous males are amongst the most vulnerable in Australian society [43-45]. Indigenous males have long recognised the significance of the loss of their authority and self-esteem through alienation, loss of culture, and recurrent challenges to individual and collective spiritual well being and a sense of control over ones life [46]. These have been considered as important antecedents to violence, self harm, drug dependence, depression, and anxiety, with suicide among young Indigenous males being an important indicator of the social, emotional and psychological well-being of Indigenous communities [45]. This particular vulnerability of Indigenous male youth resonates with the pattern of mastery exposed in this analysis.

Despite the conceptual congruence of mastery within this population, limitations are worth noting. This report is based on small numbers, but primary objectives were to explore within-population relations between psychosocial and behavioural characteristics, rather than assessment of the prevalence of such factors. Despite a potential for information bias through adapted instruments, we contend this analysis provides a reasonable assessment of the cross-cultural utility of mastery within an Indigenous settlement. The cross-sectional nature of the study precludes determination of causality and the temporal direction of the relations assessed. Reliance on volunteers could affect results through selection bias. Reporting bias is possible for self-reported data, although the extent of heterogeneity observed for all measures, and the lack of differences between the broader sample and persons completing psychosocial surveys, suggests no underlying systematic bias, and adequate representativeness. The acceptability of questionnaire items might bear improvement, however, given that 16% of participants did not fully complete the survey. While the overall sample was representative of the target population, the generalisability of the results may be limited given that probability-based sampling was not viable for the target population and hence not utilised. Few data have been reported, however, on psychosocial status in Indigenous populations, and this report provides a basis for initiating more comprehensive longitudinal surveys with representative samples of Indigenous peoples to assess relationships between perceived control, mental health and health-related behaviour across the life course.

Understanding the psychosocial correlates of health and behaviour in Indigenous peoples involves significant challenges, but offers tremendous opportunity. Assessment of mastery was deemed relevant and important by the community under study, demonstrated adequate semantic and construct equivalence and, on the whole, correlated with measures of behaviour and acute and chronic stressors in the anticipated direction. Mastery is a positive construct, which in this Indigenous Australian population seems to have utility in linking the perception of adversity to health-related behaviour. Making explicit the relationship between disadvantage and poor health in marginalised populations is requisite to the identification of factors amenable to intervention. Assessment and cross-cultural adaptation of mastery and related constructs offer the methods and opportunity to explore deeper understandings of the nature, interactions, and consequences of an individual's historical and social context, and the relationship between disadvantage and poor health status in marginalised populations. Little research has focused on the role of such factors in attempting to explain the unacceptable level of health disparity of Indigenous peoples. Ultimately, however, this work points to the start of a process of unpacking the social determinants of Indigenous peoples' health, which embraces the symbiotic relationship between the body, mind and spirit, individual and the collective, the person and place, and the past, present and future.

## Competing interests

The author(s) declare that they have no competing interests.

## Authors' contributions

MD conceived and designed the study, undertook the analyses, interpreted the results, and drafted the manuscript. AB contributed to analysis and interpretation of the data and drafting of the manuscript with attention to Indigenous cultural and scientific perspectives. GD made substantial intellectual contributions through cultural adaptation of psychosocial measures, design and implementation of culturally appropriate data collection protocols, acquisition of data, and critical revision of the manuscript for important intellectual content. MDC provided substantial contributions to analysis and interpretation of the data, and critical revision of the manuscript for important intellectual content. KOD made substantial contributions through conception and design of the study, and critical revision of the manuscript for key intellectual content. All authors read and approved the final manuscript.

## References

[B1] Adler NE, Boyce T, Chesney MA, Cohen S, Folkman S, Kahn RL, Syme SL (1994). Socioeconomic status and health: the challenge of the gradient. American Psychologist.

[B2] Marmot M (2005). Social determinants of health inequalities. Lancet.

[B3] Donkin A, Goldblatt P, Lynch K (2002). Inequalities in life expectancy by social class 1972-1999. Health Statistics Quarterly.

[B4] Australian Bureau of Statistics and the Australian Institute of Health and Welfare (2003). The Health and Welfare of Australia's Aboriginal and Torres Strait Islander Peoples 2003 (ABS Cat No. 4704.0).

[B5] Steering Committee for the Review of Government Service Provision (2003). Overcoming Indigenous Disadvantage: Key Indicators 2003.

[B6] Elstad JI (1998). The psycho-social perspective on social inequalities in health. Sociology of Health & Illness.

[B7] Pollitt RA, Daniel M, Kaufman JS, Lynch JW, Salonen JT, Kaplan GA (2005). Mediation and modification of association between hostility, hopelessness and progression of carotid atherosclerosis. Journal of Behavioral Medicine.

[B8] Siegrist J, Marmot M (2004). Health inequalities and the psychosocial environment - two scientific challenges. Social Science & Medicine.

[B9] Gump BB, Matthews KA, Raikkonen K (1999). Modeling relationships among socioeconomic status, hostility, cardiovascular reactivity, and left ventricular mass in African American and White children. Health Psychology.

[B10] Lynch JW, Kaplan GA, Cohen RD, Tuomilehto J, Salonen JT (1996). Do cardiovascular risk factors explain the relation between socioeconomic status, risk of all-cause mortality, cardiovascular mortality, and acute myocardial infarction?. American Journal of Epidemiology.

[B11] Ruberman JA, Weinblatt E, Goldberg JD, Chaudhary BS (1984). Psychosocial influences on mortality after myocardial infection. New England Journal of Medicine.

[B12] Cohen S, Kaplan GA, Salonen JT (1999). The role of psychological characteristics in the relation between socioeconomic status and perceived health. Journal of Applied Social Psychology.

[B13] Fiscella K, Franks P (1997). Does psychological distress contribute to racial and socioeconomic disparities in mortality?. Social Science & Medicine.

[B14] Gallo LC, Matthews KA (2003). Understanding the association between socioeconomic status and physical health: Do negative emotions play a role?. Psychological Bulletin.

[B15] Pearlin LI, Lieberman MA, Menaghan EG, Mullan JT (1981). The stress process. Journal of Health and Social Behavior.

[B16] Bandura A (1997). Self-Efficacy: The Exercise of Control.

[B17] Rotter JB (1975). Some problems and misconceptions related to the construct of internal versus external reinforcement. Journal of Consulting and Clinical Psychology.

[B18] Taylor SE, Seeman TE (1999). Psychosocial resources and the SES-health relationship. Annals of the New York Academy of Sciences.

[B19] Cott CA, Gignac MAM, Badley EM (1999). Determinants of self rated health for Canadians with chronic disease and disability. Journal of Epidemiology and Community Health.

[B20] Anderzen I, Arnetz BB (1999). Psychophysiological reactions to international adjustment. Results from a controlled, longitudinal study. Psychotherapy & Psychosomatics.

[B21] Dalgard OS, Lund Haheim L (1998). Psychosocial risk factors and mortality: a prospective study with special focus on social support, social participation, and locus of control in Norway. Journal of Epidemiology and Community Health.

[B22] Ross CE, Reynolds JR, Geis KJ (2000). The contingent meaning of neighborhood stability for residents' psychological well-being. American Sociological Review.

[B23] Vlahov D, Galea S, Resnick H, Ahern J, Boscarino JA, Bucuvalas M (2002). Increased use of cigarettes, alcohol, and marijuana among Manhattan, New York, residents after the September 11th terrorist attacks. American Journal of Epidemiology.

[B24] Daniel M, Cargo MD, Lifshay J, Green LW (2004). Cigarette smoking, mental health and social support: Data from a Northwestern First nation. Canadian Journal of Public Health.

[B25] Daniel M, Gamble D, Henderson J, Burgess S (1995). Diabetes prevalence, behavioural and anthropometric risk factors, and psychosocial constructs in three aboriginal communities in central British Columbia. Chronic Diseases in Canada.

[B26] O'Dea K, White N, Sinclair AJ (1988). An investigation of nutrition-related risk factors in an isolated Aboriginal community in northern Australia: advantages of a traditionally-oriented lifestyle. Medical Journal of Australia.

[B27] Maple-Brown L, Brimblecombe J, Chisholm D, O'Dea K (2004). Diabetes care and complications in a remote primary care health setting. Diabetes Research and Clinical Practice.

[B28] Rowley KG, Daniel M, Skinner M, White G, O'Dea K (2000). Effectiveness of a community-directed "healthy lifestyle" programme in a remote Australian Aboriginal community. Australian and New Zealand Journal of Public Health.

[B29] Daniel M, O'Dea K, Rowley KG, McDermott R, Kelly S (1999). Glycated hemoglobin as an indicator of social environmental stress among indigenous versus westernized populations. Preventive Medicine.

[B30] Turner RJ, Lloyd DA (1999). The stress process and the social distribution of depression. Journal of Health and Social Behavior.

[B31] McDermott R, O'Dea K, Rowley K, Knight S, Burgess P (1998). Beneficial impact of the homelands movement on health outcomes in central Australian Aborigines. Australian & New Zealand Journal of Public Health.

[B32] Daniel M, Linder GF, Breslow L (2002). Marginal people. Encyclopedia of Public Health.

[B33] Karasek R, Baker D, Marxer F, Ahlbom A, Theorell T (1981). Job decision latitude, job demands, and cardiovascular disease.  A prospective study of Swedish men.. American Journal of Public Health.

[B34] Theorell T, Alfredsson L, Knox S, Persk A, Sevensson J, Waller D (1984). On the interplay between socioeconomic factors, personality and work environment in the pathogenesis of cardiovascular disease.. Scandinavian Journal of Work and Environmental Health.

[B35] Chandler MJLC (1998). Cultural continuity as a hedge against suicide in Canada's First Nations. Transcultural Psychiatry.

[B36] Kessler RC, Mickelson KD, Williams DR (1999). The prevalence, distribution, and mental health correlates of perceived discrimination in the United States. Journal of Health and Social Behavior.

[B37] Kirmayer LBGTCL (2000). The mental health of Aboriginal peoples: Transformations of identity and community. Canadian Journal of Psychiatry.

[B38] Wilkinson RG (1999). Health, hierarchy, and social anxiety. Annals of the New York Academy of Sciences.

[B39] Brunner E, Davey Smith G, Marmot M, Canner R, Beksinska M (1996). Childhood social circumstances and psychosocial and behavioural factors as determinants of plasma fibrinogen. Lancet.

[B40] Lynch JW, Kaplan GA, Shema SJ (1997). Cumulative impact of sustained economic hardship on physical, cognitive, psychological and social functioning. New England Journal of Medicine.

[B41] Eaton WW, Armenian H, Gallo J, Pratt L, Ford DE (1996). Depression and risk for onset of type II diabetes: a prospective population-based study. Diabetes Care.

[B42] Anda R, Williamson D, Jones D, Macera C, Eaker E, Glassman A, Marks J (1993). Depressed affect, hopelessness, and the risk of ischemic heart disease in a cohort of U.S. adults. Epidemiology.

[B43] Swann P, Raphael B (1995). Ways Forward. National Consultancy Report on Aboriginal and Torres Strait Islander Mental Health.

[B44] Moon L, Meyer P, Grau J (1999). Australia's young people: their health and wellbeing 1999. AIHW Cat. No. PHE 19..

[B45] Hunter E, Reser J, Baird M, Reser P (1999). An analysis of suicide in Indigenous communities of North Queensland: The historical, cultural and symbolic landscape. Report for the Mental Health/Health Services Development Branch, Commonwealth Department of Health and Aged Care.

[B46] Spry F, Lowe H (2001). Inquiry into the Education of Boys - Submission from the Northern Territory Aboriginal Male Health Reference Committee (NTAMHRC) to the Parliament of Australia House of Representatives Standing Committee on Employment, Education and Workplace Relations.

